# Triglyceride Polygenic Score Identifies Individuals Who May Respond Differently to Aspirin in Primary Prevention

**DOI:** 10.1002/cpt.70446

**Published:** 2026-08-14

**Authors:** Peter D. Fransquet, Chenglong Yu, Cammie Tran, Sultana Monira Hussain, Chad A. Bousman, Mark R. Nelson, Andrew M. Tonkin, John J. McNeil, Paul Lacaze

**Affiliations:** ^1^ School of Public Health and Preventive Medicine Monash University Melbourne Victoria Australia; ^2^ Victorian Heart Institute Monash University Melbourne Victoria Australia; ^3^ Department of Medical Genetics, Physiology & Pharmacology, and Psychiatry University of Calgary Calgary Alberta Canada; ^4^ Menzies Institute for Medical Research University of Tasmania Dynnyrne Tasmania Australia

## Abstract

Low‐dose aspirin is no longer routinely recommended for the primary prevention of cardiovascular disease in older adults due to a lack of net benefit over bleeding risk. We hypothesized that genetic subgroups may experience differential harm or benefit from aspirin therapy. To investigate this, we screened 572 polygenic scores (PGSs) for modification of aspirin's effect on major bleeding and major adverse cardiovascular events (MACE) in the Aspirin in Reducing Events in the Elderly (ASPREE) randomized, placebo‐controlled trial of daily 100 mg aspirin. Participants were aged ≥70 years (≥65 years for US minorities) and free of cardiovascular disease, dementia, or physical disability at enrolment. Among participants with high‐quality genotyping data (*n* = 13,571), PGS–aspirin interactions were tested using Cox proportional hazards models with Bonferroni correction for multiple testing. During a median follow‐up of 4.6 years, 414 major bleeding events and 464 MACE occurred. A triglyceride‐related PGS (PGS003144) significantly modified aspirin‐associated bleeding (interaction *P* = 5.9 × 10^−5^; Bonferroni‐adjusted *P* = 0.034). In the lowest quintile of the PGS distribution, aspirin increased major bleeding risk (HR 2.28; 95% CI: 1.45–3.58; *P* = 0.00036), including separate bleeding subgroups gastrointestinal (HR 3.17; 95% CI: 1.48–6.80; *P* = 0.0029), and intracranial bleeding (HR 4.10; 95% CI: 1.54–11.0; *P* = 0.0049). In contrast, in the highest quintile, aspirin was associated with lower risk of bleeding (HR 0.62; 95% CI 0.38–0.97) and reduced MACE (HR 0.66; 95% CI 0.44–0.99). Baseline serum triglycerides showed similar effect modification. These hypothesis‐generating findings suggest triglyceride‐related genetic variation may identify individuals with differential responses to aspirin.

## Graphical abstract

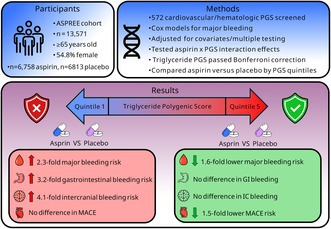

**Study Highlights**

**WHAT IS THE CURRENT KNOWLEDGE ON THE TOPIC?**

Low‐dose aspirin is no longer routinely recommended for primary prevention in older adults because randomized trials have shown that bleeding risks outweigh cardiovascular benefits at the population level. However, these recommendations do not account for interindividual biological or genetic differences that may influence aspirin response, and reliable tools to identify subgroups with a favorable benefit–risk profile are lacking.

**WHAT QUESTION DID THIS STUDY ADDRESS?**

This study tested whether polygenic scores (PGSs) related to cardiovascular and hematologic traits modify the effects of low‐dose aspirin on major bleeding and major adverse cardiovascular events (MACE) in older adults enrolled in the ASPREE randomized trial.

**WHAT DOES THIS STUDY ADD TO OUR KNOWLEDGE?**

A triglyceride‐related PGS identified marked heterogeneity in aspirin response. Individuals with genetically lower triglyceride predisposition experienced increased major bleeding without cardiovascular benefit, whereas those with higher genetic triglyceride burden had lower risks of both bleeding and MACE with aspirin. Measured triglyceride levels showed a concordant pattern, supporting biological plausibility.

**HOW MIGHT THIS CHANGE CLINICAL PHARMACOLOGY OR TRANSLATIONAL SCIENCE?**

While hypothesis generating, these findings suggest that genetically informed stratification could refine aspirin use for primary prevention by identifying individuals likely to be harmed vs. those who may benefit. Incorporating lipid‐related genetic markers into risk assessment frameworks may advance precision pharmacotherapy and support future trials of genotype‐guided aspirin prescribing in older adults.

Low‐dose aspirin has historically been widely used for the primary prevention of atherothrombotic cardiovascular disease events in older adults. However, recent evidence has tempered its use in this setting due to increased bleeding risk and limited vascular benefit.^1^, ^2^, ^3^, ^4^ Large randomized trials, including ARRIVE, ASCEND, and ASPREE,^3^, ^5^, ^6^ reported unfavorable benefit‐harm ratios for aspirin in primary prevention, largely driven by excess bleeding events. These trials, however, did not account for interindividual variability in aspirin response, including genetic factors that may influence susceptibility to bleeding or cardiovascular events. Identifying biological determinants of differential aspirin response could provide a pathway toward more personalized therapeutic use. Supporting this concept, our previous work in the ASPREE trial population demonstrated that individuals with specific lipoprotein(a) genotypes experienced net benefit from low‐dose aspirin in primary prevention,^7^ providing proof‐of‐concept for genetically informed risk stratification.

Genetic variation is known to contribute to variability in drug response, and several biological processes, including lipid metabolism, platelet function, and vascular integrity,^8^, ^9^, ^10^, ^11^ may influence aspirin effects. Polygenic scores (PGSs), which aggregate the effects of many different common genetic variants associated with a trait or disease, can capture complex genetic contributions to cardiovascular and metabolic outcomes.^12^ PGSs may therefore help identify biological variation relevant to aspirin‐related bleeding risk. Although prior studies suggest that genetic predisposition may modify aspirin's effect on cardiovascular outcomes,^13^, ^14^ the role of PGSs in predicting aspirin‐associated bleeding events remains largely unexplored.

In this post hoc genetic analysis of the ASPREE randomized trial, we systematically screened 572 PGSs related to cardiovascular, coagulation, and hematologic traits to evaluate whether polygenetic factors modify aspirin response. We hypothesized that specific PGSs would interact with aspirin allocation to identify individuals at increased bleeding risk. We also examined whether PGSs modify aspirin's effect on major adverse cardiovascular events (MACE). Our objective was to identify genetic factors that influence both bleeding and cardiovascular risk associated with aspirin in the primary prevention setting.

## MATERIALS AND METHODS

### Study design and population

We conducted a post hoc genetic analysis of participants in the ASPREE trial, a randomized, placebo‐controlled study of low‐dose aspirin (100 mg of enteric‐coated aspirin or matching placebo) in healthy older adults.^15^ Participants were aged ≥70 years from Australia and the United States (≥65 years for US minorities), with no prior cardiovascular disease, dementia, physical disability, or chronic illness likely to limit survival to <5 years. Recruitment was conducted between March 2010 and December 2014. All participants provided written informed consent. The trial was approved by institutional review boards at all sites and registered at ClinicalTrials.gov (NCT01038583).

### End points

The primary outcome was major bleeding, defined as a composite of hemorrhagic stroke, symptomatic intracranial bleeding, or clinically significant extracranial bleeding leading to transfusion, hospitalization, surgery, or death. The secondary outcome was MACE (fatal or non‐fatal ischemic stroke, non‐fatal myocardial infarction, or coronary heart disease death). Gastrointestinal bleeding and intracranial bleeding were additionally examined in bleeding subtype analyses to further characterize the major bleeding outcome. Outcomes were adjudicated by committees of clinical experts blinded to treatment allocation.

### Genotyping and polygenic scores

Baseline DNA was genotyped using the Axiom 2.0 Precision Medicine Diversity Array. Standard quality control steps were applied,^16^, ^17^ and imputation was performed using the TOPMed reference panel GRCh38 (hg38).^18^, ^19^, ^20^ PGSs were obtained from the PGS Catalog for five trait categories: cardiovascular disease, cardiovascular measurement, hematologic measurement, inflammatory measurement, and lipid/lipoprotein measurement available up until September 22, 2025.^21^, ^22^ PGSs were generated using PLINK 1.9,^23^ and scores with low quality control metrics were excluded from the analysis. These included scores with low SNP availability (high data missingness), scores which did not use samples of European ancestry in genome‐wide association studies, and scores with detected issues related to distributional quality, whereby the approximate PGS distribution deviated substantially from a normal distribution (for details, see Supplementary Material S1).

### Statistical and exploratory analysis

For each PGS, Cox proportional hazards models independently assessed interactions with aspirin allocation and major bleeding events, adjusted for age, sex, and the first 10 genetic principal components. To assess whether interaction signals clustered within specific biological domains, trait‐group enrichment analyses were performed. PGSs were grouped into broad biological categories based on their reported trait. Enrichment of nominally significant aspirin–PGS interactions (*P* < 0.05) within each category was assessed using Fisher's exact tests, comparing the observed number of significant interactions with the number expected based on category representation among all tested PGSs. *P* values for interactions were Bonferroni‐corrected, and significant PGSs were further analyzed with additional adjustment for baseline smoking, alcohol use, body mass index (BMI), hypertension, diabetes, chronic kidney disease (CKD), and prior aspirin or anti‐inflammatory use. Analyses were conducted using complete‐case data. Participants with missing covariate information required for a given model were excluded from that analysis, and no imputation of missing data was performed. Distribution‐based quintile‐specific analyses compared aspirin vs. placebo effects for major bleeding, MACE, and bleeding subtypes. To assess whether PGS modification of aspirin response differed by sex, a three‐way interaction term between treatment allocation, PGS003144, and sex was examined. Baseline characteristics were compared across PGS quintiles using chi‐square and ANOVA. Analyses were performed in R version 4.5.1. Exploratory analysis was performed based on results of polygenic score screening.

## RESULTS

### Polygenic score screening

**Figure 1:**
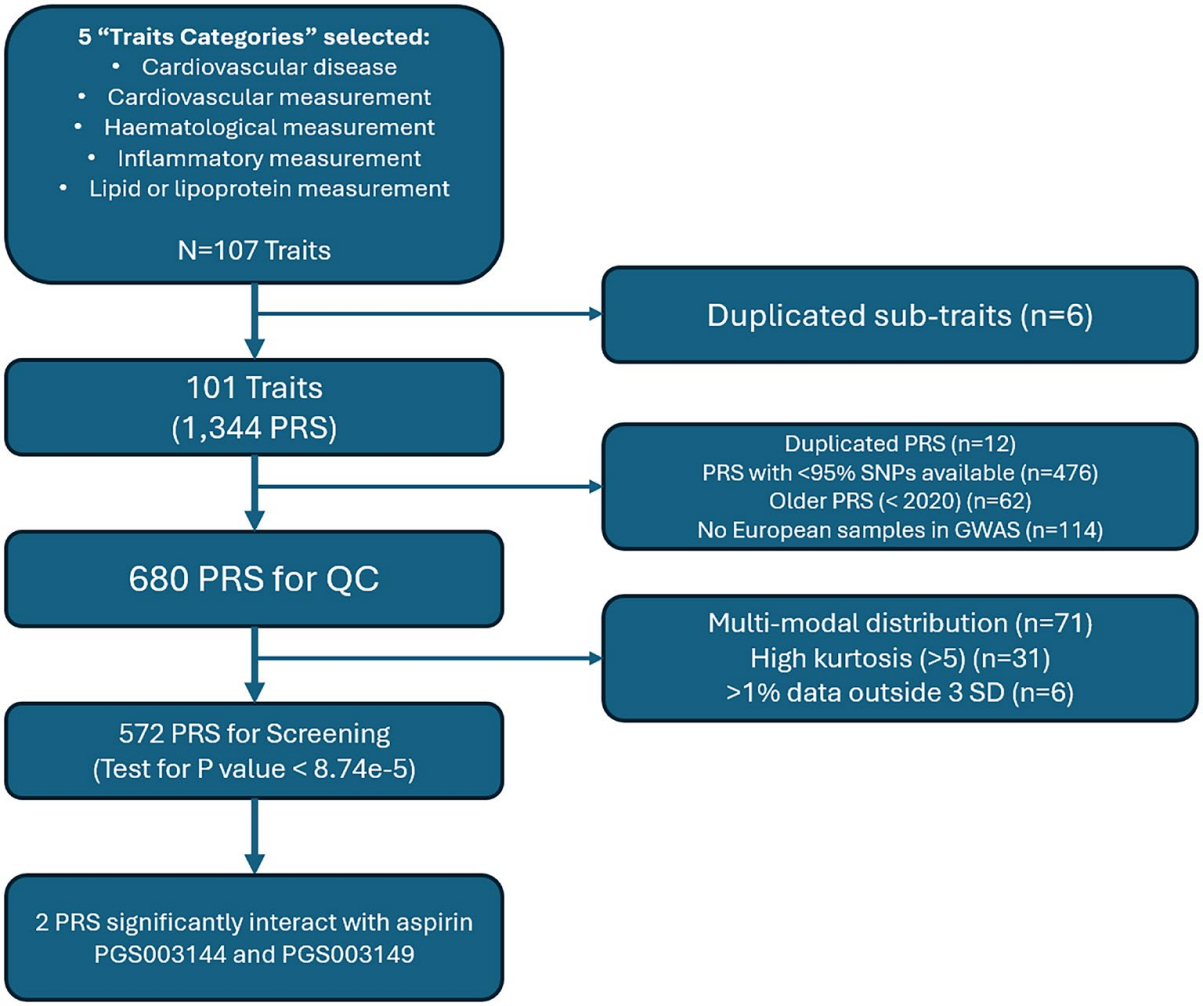
Flow diagram for polygenic score selection for subsequent screening.

Of 1,344 polygenic scores derived from 101 traits across five biologically relevant categories, 572 remained after quality control (**Figure** **1**). Two triglyceride‐related PGSs (PGS003144 and PGS003149) showed a significant interaction with aspirin allocation for major bleeding events after Bonferroni correction (adjusted *P* = 0.034 and *P* = 0.039, respectively). These scores were derived from the same source study of 9,592 individuals and were highly correlated in the ASPREE cohort (r = 0.96, *P* < 0.0001), indicating that they capture largely the same underlying triglyceride‐related genetic signal.^24^ PGS003144, which demonstrated the strongest interaction signal and included a slightly larger number of variants, was selected for detailed follow‐up analyses. For PGS003144, each one standard deviation increase in genetically predicted triglyceride levels was associated with attenuation of aspirin‐associated bleeding risk (interaction HR = 1.17, 95% CI 1.01–1.36; interaction *P* = 5.88 × 10^−5^; adjusted *P* = 0.034). To determine whether interaction signals clustered within specific biological domains, we examined enrichment of nominally significant aspirin–PGS interactions. Among the 572 tested PGSs, 91 demonstrated nominal evidence of interaction with aspirin allocation for major bleeding (*P* < 0.05). Triglyceride‐related scores were strongly enriched among these interaction signals. Although triglyceride scores represented only 20 of 572 tested PGSs (3.5%), 18 of the 91 nominally significant interactions (19.8%) were triglyceride‐related. This represented a 5.7‐fold enrichment compared with expectation (OR = 58.4, Fisher's exact *P* = 1.3 × 10^−13^; FDR = 2.3 × 10^−12^). No other lipid‐related trait category demonstrated comparable enrichment (**Table** **S1**).

### Study population

A total of 13,571 genotyped ASPREE participants were included in the analysis. The mean age was 75 years (SD 4.3) and 54.8% were female. Baseline characteristics of the participants, stratified by PGS003144 quintiles are presented in **Table** **1**. Overall, 6,758 participants (49.8%) received aspirin and 6,813 (50.2%) received placebo, with median follow‐up of 4.6 years (interquartile range (IQR)). Baseline differences across PGS quintiles included BMI (*P* = 0.011), hypertension (*P* = 0.005), diabetes (*P* < 0.0001), CKD (*P* = 0.004), alcohol use (*P* < 0.001), lipid levels (all P < 0.001), and statin use (P < 0.001).

**Table 1:** Baseline characteristics and trial period endpoints across quintiles

Baseline characteristics	All participants (No. = 13,571)	Quintile 1 (No. = 2,715)	Quintiles 2–4 (No. = 8,142)	Quintile 5 (No. = 2,714)	*P* value
Age, mean (SD)	75 (4.3)	75.1 (4.4)	75 (4.2)	74.8 (4.2)	0.053
Sex female, No. (%)	7,441 (54.8)	1,473 (54.3)	4,462 (54.8)	1,506 (55.5)	0.66
BMI, mean (SD)	28 (4.6)	27.8 (4.6)	28.1 (4.6)	28.1 (4.5)	**0.011**
Hypertension, No. (%)	9,993 (73.6)	1993 (73.4)	6,038 (74.2)	2022 (74.5)	**0.005**
Diabetes, No. (%)	1,321 (9.7)	194 (7.2)	796 (9.8)	331 (12.2)	**<0.0001**
CKD, No. (%)	3,256 (24)	610 (22.5)	1935 (23.8)	711 (26.2)	**0.004**
Smoking status, No. (%)					
Current/Former	6,022 (44.4)	1,193 (43.9)	3,599 (44.2)	1,230 (45.3)	0.52
Never	7,549 (55.6)	1,522 (56.1)	4,543 (55.8)	1,484 (54.7)	
Alcohol use, No. (%)					
Current	10,702 (78.9)	2,221 (81.8)	6,369 (78.2)	2,112 (77.8)	**0.0001**
Former/Never	2,869 (21.1)	494 (18.2)	1773 (21.8)	602 (22.2)	
Lipids					
HDL mmol/L, median (IQR)^a^	1.5 (0.5)	1.6 (0.6)	1.5 (0.5)	1.4 (0.5)	**<0.0001**
LDL mmol/L, median (IQR)^b^	3 (1.1)	3.1 (1.1)	3 (1.1)	3 (1.4)	**<0.0001**
Triglycerides mmol/L, median (IQR)^c^	1.2 (0.7)	1 (0.6)	1.2 (0.7)	1.5 (0.9)	**<0.0001**
Medication					
Statins, No. (%)	4,180 (30.8)	636 (23.4)	2,472 (30.4)	1,072 (39.5)	**<0.0001**
NSAID, No. (%)	1795 (13.2)	336 (12.4)	1,093 (13.4)	366 (13.5)	0.34
Previous aspirin use, No. (%)	1,300 (9.6)	247 (9.1)	789 (9.7)	264 (9.7)	0.63
Trial aspirin allocation, No. (%)	6,758 (49.8)	1,342 (49.4)	4,020 (49.4)	1,396 (51.4)	0.16
Endpoints, No. (%)					
Major bleeding	414 (3.1)	87 (3.2)	249 (3.1)	78 (2.9)	0.78
MACE	464 (3.4)	80 (2.9)	288 (3.5)	96 (3.5)	0.31
Gastrointestinal bleeding	169 (1.3)	35 (1.3)	102 (1.3)	32 (1.2)	0.093
Intercranial bleeding	112 (0.8)	26 (0.96)	70 (0.86)	16 (0.59)	0.28

BMI, body mass index; CKD, chronic kidney disease; HDL, high‐density lipoprotein; LDL, low‐density lipoprotein; MACE, major adverse cardiovascular events; NSAID, non‐steroidal anti‐inflammatory drug use.

^a^ Data on 341 participants missing.

^b^ Data on 385 participants missing.

^c^ Data on 159 participants missing.

### Continuous genetic risk analysis

To evaluate whether the observed interaction was dependent on arbitrary quintile thresholds, PGS003144 was analyzed as a continuous variable using spline modeling. The association demonstrated a graded attenuation of aspirin‐associated bleeding risk across the triglyceride PGS distribution, with the highest bleeding risk observed among participants with low genetically predicted triglyceride levels and little evidence of excess bleeding among those with higher genetic scores (**Figure** **S1**). Similar directional patterns were observed for MACE, although the associated confidence intervals were wider (**Figure** **S2**). No evidence of sex‐specific modification of the aspirin–PGS interaction was observed (Aspirin × PGS003144 × Sex interaction HR = 1.04, *P* = 0.865). To assess whether the observed interaction was independent of measured triglyceride levels, an additional sensitivity analysis was performed adjusting for baseline triglyceride concentrations in fully adjusted statistical models. The aspirin × PGS003144 interaction remained essentially unchanged (interaction *P* = 5.28 × 10^−5^), suggesting that the genetic score captures information beyond measured triglyceride levels alone.

### Genetic stratification

In the lowest quintile of the PGS003144 triglyceride PGS distribution (Q1), aspirin use vs. placebo was associated with a markedly higher risk of major bleeding (HR 2.28; 95% CI: 1.45–3.58; *P* = 0.00036). Follow‐up analyses of bleeding subtypes also demonstrated increased risks of both gastrointestinal bleeding (HR 3.17; 95% CI: 1.48–6.80; *P* = 0.0029) and intracranial bleeding (HR 4.10; 95% CI: 1.54–11.0; *P* = 0.0049) after adjusting for multiple covariates (**Table** **2**, basic model **Table** **S2**). However, no association was observed in Q1 for MACE (HR 1.04; 95% CI: 0.67–1.62; *P* = 0.86) in individuals randomized to aspirin vs. placebo.

**Table 2:** Outcome risk of aspirin vs. placebo, within PGS003144 polygenic score quintile stratification, fully adjusted model

Outcome	Polygenic score quintile 1				Polygenic score quintile 5			
	No. of events	HR	95% CI	*P* value*	No. of events	HR	95% CI	*P* value*
Major bleeding	87	2.28	1.45–3.58	**0.00036**	78	0.62	0.38–0.97	**0.038**
MACE	80	1.04	0.67–1.62	0.86	95	0.66	0.44–0.99	**0.0497**
Gastrointestinal bleeding	35	3.17	1.48–6.80	**0.0029**	32	0.71	0.35–1.43	0.33
Intercranial bleeding	26	4.10	1.54–11.0	**0.0049**	16	0.37	0.13–1.11	0.079

CI, confidence interval; HR, hazard ratio; MACE, major adverse cardiovascular events.

* Adjusted for age, sex, smoking, alcohol consumption, body mass index, previous regular aspirin use, hypertension, diabetes, chronic kidney disease, non‐steroidal anti‐inflammatories, top 10 principal components.

**Figure 2:**
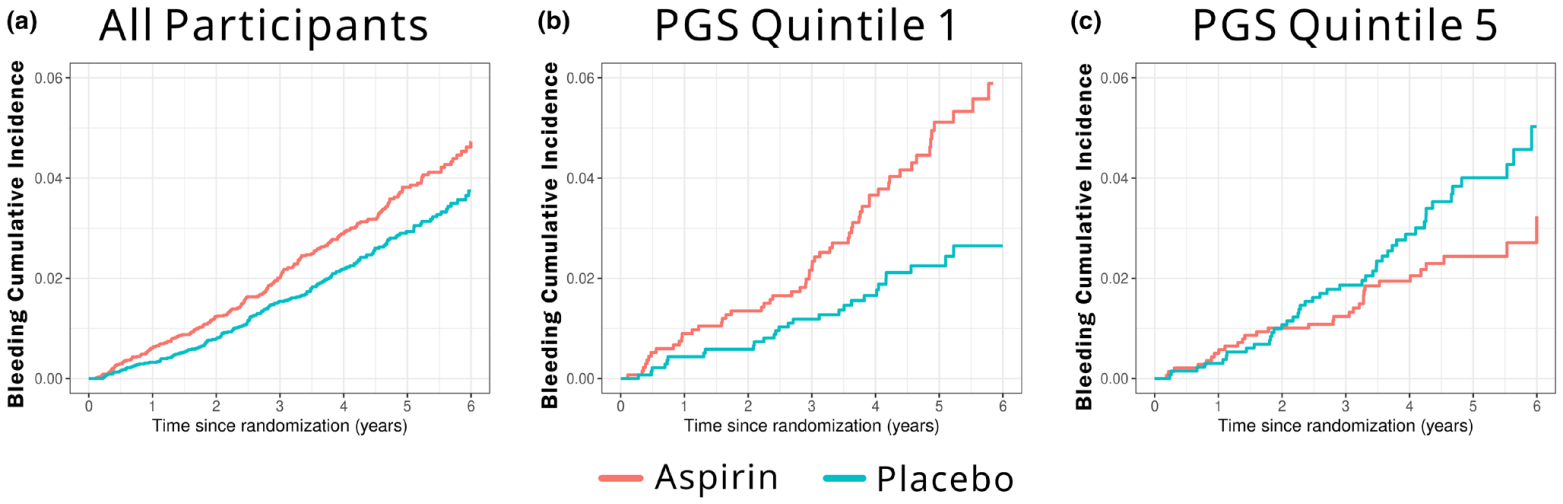
Aspirin use and 6‐year cumulative incidence for major bleeding in all participants and within PGS003144 genetic quintiles. (**a**) All participants: total aspirin group = 5,831, MH in aspirin group = 227; total placebo group = 5,907, MH in placebo group = 178. (**b**) Triglyceride genetic quintile 1: total aspirin group = 1,149, MH in aspirin group = 57; total placebo group = 1,193, MH in placebo group = 28. (**c**) Triglyceride genetic quintile 5: total aspirin group = 1,213, MH in aspirin group = 31; total placebo group = 1,158, MH in placebo group = 45. PGS, polygenic score.

In contrast, participants in the highest PGS quintile (Q5) randomized to aspirin vs. placebo had lower risk of major bleeding (HR 0.62; 95% CI: 0.38–0.97; *P* = 0.038) and MACE (HR 0.66; 95% CI: 0.44–0.99; *P* = 0.0497), with no significant effect on gastrointestinal or intracranial bleeding. Six‐year cumulative incidence of bleeding, stratified by aspirin and placebo allocation, for (a) all participants, (b) PGS quintile 1, and (c) PGS quintile 5, can be seen in **Figure** **2** (MACE results in **Figure** **S3**).

### Clinical risk differences

Absolute risk estimates demonstrated meaningful differences across triglyceride PGS strata (**Table** **S3**). In the lowest quintile (Q1), major bleeding occurred in 4.4% of aspirin‐treated participants compared with 2.0% of placebo‐treated participants, corresponding to an absolute risk increase of 2.36% and an estimated number‐needed‐to‐harm (NNH) of 42 over a median follow‐up of 4.7 years. In contrast, MACE incidence was similar between treatment groups (3.0% vs. 2.9%; absolute risk difference 0.07%).

In the highest quintile (Q5), major bleeding occurred in 2.3% of aspirin‐treated participants compared with 3.5% of placebo‐treated participants, corresponding to an absolute risk reduction of 1.20% and an estimated number‐needed‐to‐treat (NNT) of 83 over a median follow‐up of 4.7 years. Similarly, MACE occurred in 2.9% of aspirin‐treated participants compared with 4.2% of placebo‐treated participants, corresponding to an absolute risk reduction of 1.24% and an estimated NNT of 81.

### Exploratory analyses

Baseline fasting triglyceride levels were available for all participants of the ASPREE trial. Upon exploratory analysis of these measured triglyceride levels, we found moderate correlation between measured levels and the PGS003144 triglyceride PGS (*r* = 0.30; *P* < 0.0001). Participants in the lowest measured triglyceride quintile (Q1) and assigned to aspirin had a higher risk of major bleeding (HR 1.68; 95% CI: 1.12–2.51; *P* = 0.01) and intracranial bleeding (HR 3.10; 95% CI: 1.39–6.91; *P* = 0.006) when compared to placebo. No differences were observed in the other measured triglyceride‐level quintiles (**Table** **3**).

**Table 3:** Aspirin vs. placebo stratified by triglyceride‐level quintiles

Outcome	Quintile 1—low triglyceride levels (0.2–0.8 mmolL)				Quintile 5—high triglyceride levels (1.7–9.4 mmolL)			
	No. of events	HR	95% CI	*P* value*	No. of events	HR	95% CI	*P* value*
Major bleeding	100	1.68	1.12–2.51	**0.01**	85	0.92	0.60–1.41	0.68
MACE	64	0.79	0.48–1.28	0.33	120	0.77	0.53–1.10	0.15
Gastrointestinal bleeding	38	1.40	0.74–2.68	0.30	38	1.59	0.83–3.05	0.16
Intercranial bleeding	32	3.10	1.39–6.91	**0.006**	17	0.56	0.21–1.51	0.25

* Adjusted for age, sex, and baseline statin use.

## DISCUSSION

In this post hoc genetic analysis of 13,571 participants from the ASPREE randomized control trial, a triglyceride‐level PGS identified differential responses to aspirin for major bleeding and MACE. Participants in the lowest quintile of the triglyceride PGS had increased risk of major bleeding, gastrointestinal and intracranial bleeding with aspirin, whereas those in the highest quintile experienced lower major bleeding risk and a 1.5‐fold reduction in MACE. These findings suggest that genetic variation in triglyceride‐related pathways (and in measured triglyceride levels) may be associated with differential bleeding and cardiovascular risk with aspirin in the primary prevention setting. However, as these analyses were post hoc, independent replication will be required before any clinical implications can be considered.

The magnitude of these differences may also be clinically meaningful. In the lowest triglyceride PGS quintile, aspirin was associated with one additional major bleeding event for approximately every 42 individuals treated, with little corresponding reduction in MACE. Conversely, in the highest quintile, aspirin was associated with reductions in both major bleeding and MACE, corresponding to NNT values of approximately 80 for each outcome. While these estimates should be interpreted cautiously given the post hoc nature of these analyses, they illustrate the potential clinical relevance of genetic stratification of aspirin response. Overall, the evidence was stronger and more consistent for major bleeding than for MACE, and the cardiovascular findings should therefore be interpreted cautiously pending independent replication.

Our findings add a new dimension to the ongoing debate over aspirin use for primary prevention of cardiovascular disease in older adults. Although aspirin has long been recognized for its cardioprotective effects, recent evidence has suggested that the harms from bleeding outweighs any vascular benefit in primary prevention.^3^, ^5^, ^6^ This has resulted in the US Preventive Services Task Force (USPSTF) recommending against routine aspirin use for primary prevention in adults aged 60 years or older.^25^ However, the above trials reflect an overall harm‐to‐benefit ratio calculated in all trial participants, without stratification or subgrouping. The USPSTF population‐level guidance does not account for potential interindividual variation (e.g., genetic) in aspirin response.

Our results suggest that genetic factors related to triglyceride biology may identify subgroups with differing benefit‐to‐harm profiles for aspirin therapy. If replicated in independent populations, these findings may support future investigation of more individualized approaches to aspirin use in the primary prevention setting. Previously, studies have identified possible subgroups with differential bleeding or cardiovascular risks on aspirin, based on analysis of traditional clinical risk factors rather than genetic.^26^ Our study now adds to this, specifically investigating the role of genetics in the aspirin response. Furthermore, our previous study also identified different genetic factors (specific lipoprotein(a) genotypes) interacting with aspirin for MACE reduction. We now report genetic variation related to triglyceride levels that appears to identify differential harm and bleeding risk. Combining previous findings with our current data suggests that lipid‐related genotypes and lipid metabolism have influence in differential aspirin response, both with regards to cardiovascular benefits and bleeding harms. These findings raise the possibility that genetically informed approaches to aspirin therapy could be explored in future studies, although substantial further validation would be required before clinical implementation. Notably, triglyceride‐related scores were highly enriched among nominally significant aspirin interaction signals, suggesting that the observed findings are unlikely to represent an isolated false‐positive association from a single PGS and rather reflect a broader triglyceride‐related genetic signal.

Triglycerides play a central role in the transport and storage of fatty acids in plasma and tissues.^27^ They are a key component of the lipid profile and have been primarily studied in the context of cardiovascular risk or malnutrition, with elevated levels linked to atherosclerosis and ischemic events.^28^ Fewer studies, however, have examined the relationship between low triglyceride levels and hemorrhagic outcomes. Epidemiologic evidence indicates that low triglyceride levels (or hypotriglyceridemia) (<50 mg/dL) are associated with increased risk of hemorrhagic stroke, particularly among men, individuals with hypertension, and those with low cholesterol levels.^29^ Similarly, low measured triglyceride levels have been linked to intracerebral hemorrhage and deep or infratentorial cerebral microbleeds, independent of high‐density lipoprotein or low‐density lipoprotein cholesterol levels.^30^ However, low measured triglyceride levels in older people may typically reflect age‐related changes, such as frailty,^31^, ^32^ or related malnutrition. A low polygenic score for triglycerides, by contrast, reflects an underlying genetic propensity from birth, toward lower triglyceride levels. Our findings suggest that triglyceride‐related genetic pathways may be associated with differential bleeding and cardiovascular responses to aspirin, although the underlying biological mechanisms remain uncertain.

Conversely, hypertriglyceridemia has been associated with enhanced platelet reactivity and reduced responsiveness to aspirin in patients with ischemic stroke or diabetes.^33^, ^34^ This pattern aligns with our observation that genetically higher triglyceride levels were associated with lower bleeding risk but also with reduced MACE on aspirin, implying a complex relationship between triglyceride metabolism, vascular stability, and aspirin pharmacodynamics.^35^, ^36^, ^37^ The biological mechanisms underlying this association remain uncertain and were not directly assessed in this study. Although previous work has linked triglyceride levels with platelet reactivity and aspirin responsiveness, these pathways do not fully explain the simultaneous observation of reduced bleeding and reduced MACE among participants with higher genetically predicted triglyceride levels. The observed associations may therefore reflect broader lipid‐related biological pathways, vascular biology, or other mechanisms that require further investigation.

Our exploratory analysis of measured triglyceride levels provided complementary evidence of our genetic findings: participants with low measured triglyceride levels assigned to aspirin had a higher risk of major and intracranial bleeding, whereas those with high measured triglyceride levels did not. The moderate correlation between measured and genetically predicted triglycerides (r = 0.30) supports overlapping biology, but also indicates that the PGS genetic measure captures only part of the variability in circulating triglyceride levels, which are also largely affected by dietary and other lifestyle factors, and pharmacological therapies in addition to genetic predisposition. Accordingly, the observed associations may reflect broader lipid‐related biological pathways beyond measured triglyceride concentrations alone. Importantly, the aspirin × PGS interaction remained significant after adjustment for measured triglyceride levels and conventional clinical risk factors, suggesting that triglyceride‐related genetic predisposition captures information not fully reflected by measured triglyceride levels and conventional clinical risk factors.

### Strengths and limitations

This study has several strengths, including the large, well‐characterized, randomized trial population; systematic screening of polygenic scores; rigorous ascertainment and adjudication of outcomes by expert committees masked to randomized treatment allocation; and comprehensive genetic data enabling polygenic screening. Nonetheless, limitations should be acknowledged. The analysis was post hoc and hypothesis‐generating, and the findings require replication in independent cohorts before any clinical application can be considered. Although Bonferroni correction, continuous interaction modeling, and PGS enrichment analyses supported the robustness of the triglyceride‐related signal, screening a large number of polygenic scores is associated with an inherent possibility of false‐positive findings and post‐selection inflation of effect estimates. Thus, the observed effect sizes should be interpreted cautiously and externally validated where possible. Furthermore, although follow‐up analyses were adjusted for major cardiovascular and bleeding‐related risk factors, baseline characteristics differed across triglyceride PGS quintiles and residual confounding cannot be excluded. The ASPREE cohort was predominantly of European ancestry, which may limit generalizability. In addition, while polygenic scores capture cumulative genetic risk from common alleles, they do not directly identify rare variants, causal variants, or explanatory genetic mechanisms.

## CONCLUSIONS

A triglyceride‐related PGS was associated with modification of aspirin‐associated bleeding and MACE risk in older adults and identified subgroups with differing absolute risks of bleeding and cardiovascular events. These results provide preliminary evidence that triglyceride‐related genetic variation may influence aspirin response. However, given the post hoc nature of the analyses, external validation and formal assessment of clinical utility will be required before genotype‐guided aspirin therapy could be considered in practice. Further investigation in more diverse additional populations and randomized trials is warranted to validate these findings.

## FUNDING

The ASPREE (ASPirin in Reducing Events in the Elderly) trial Biobank is supported by a Flagship cluster grant (including the Commonwealth Scientific and Industrial Research Organisation, Monash University, Menzies Research Institute, Australian National University, University of Melbourne); and a grant (5U01AG29824‐02) from the National Cancer Institute at the National Institutes of Health; and Monash University. The ASPREE project is supported by grants (U01AG029824 and U19AG062682) from the National Institute on Aging and the National Cancer Institute at the National Institutes of Health; and by grants (334047 and 1127060) from the National Health and Medical Research Council of Australia; and by Monash University and the Victorian Cancer Agency. P.L. is supported by a National Heart Foundation Future Leader Fellowship (107171) and National Health and Medical Research Council of Australia Investigator Grant (2026325). C.Y. is supported by a Vanguard Grant (108071‐2024_VG) from the National Heart Foundation of Australia. Funders were not involved in this studies design, conduct, or reporting.

## CONFLICT OF INTEREST

The authors declared no competing interests for this work.

## AUTHOR CONTRIBUTIONS

P.D.F., P.L., and C.Y. designed the research. P.D.F. performed the research and analyzed the data. All authors including, P.D.F., C.Y., C.T., S.M.H., C.A.B., M.R.N., A.M.T., J.J.M., and P.L., wrote the manuscript.

## Supporting information


Figure S1.

## Data Availability

Due to the nature of genomics, the de‐identified data we analyzed are not publicly available, but requests to the corresponding author for the data will be considered on a case‐by‐case basis.
